# Results from Lithuania’s 2018 Report Card on Physical Activity for Children and Youth

**DOI:** 10.3390/ijerph16234710

**Published:** 2019-11-26

**Authors:** Saulius Sukys, Arunas Emeljanovas, Rita Gruodyte-Raciene, Brigita Mieziene, Laima Trinkuniene, Renata Rutkauskaite, Mark Tremblay

**Affiliations:** 1 Department of Physical and Social Education, Lithuanian Sports University, Sporto 6, LT-44221 Kaunas, Lithuania; arunas.emeljanovas@lsu.lt (A.E.); rita.gruodyte@lsu.lt (R.G.-R.); brigita.mieziene@lsu.lt (B.M.); laima.trinkuniene@lsu.lt (L.T.); renata.rutkauskaite@lsu.lt (R.R.); 2 Healthy Active Living and Obesity (HALO) Research Group, Children’s Hospital of Eastern Ontario Research Institute, Ottawa, ON K1H 8L1, Canada; mtremblay@cheo.on.ca

**Keywords:** children physical activity, international comparison, Global Matrix

## Abstract

The Global Matrix 3.0 “Report Card” assessment of physical activity was developed to achieve a better understanding of the global variability in child and youth physical activity. Lithuania joined the Global Matrix 3.0. The aim of this article is to summarize the results of the first Lithuanian Report Card, which included 10 indicators, as representative of individual behaviors, sources, and settings of influence indicators, and a health-related characteristic. The grades for each indicator were based on the best available Lithuanian data. The findings showed poor Overall Physical Activity, Active Transportation (C−), and Family and Peers (D). Sedentary behavior was graded C−, and Organized Sport Participation, Community and Environment, and Government were graded C. Physical Fitness and School indicators received the highest grade (C+). The first Lithuanian Report Card on Physical Activity of Children and Youth shows that Lithuanian children and youth have less than satisfactory levels of organized physical activity, active transportation to and from school, community and built environments, and government strategies and investments. The low levels of support from family and peers require more attention from health promoters. There is a gap in the evidence about active play that should be addressed by researchers and policy makers.

## 1. Introduction

During the past several decades, physical activity has been investigated mainly with a primary focus on its health benefits. The long-term health benefits of physical activity include healthy muscles and bones, reduced risk of developing chronic diseases, better overall fitness, and a longer “health span” [1,2,3,4]. Among school-aged children in particular, physical activity is related to many health benefits in the physical, psychological, social [5], and cognitive domains [5,6,7].

The World Health Organization [8] recommends at least 1 h of moderate-to-vigorous-intensity physical activity (MVPA) per day for children and adolescents to achieve the physical activity-related health benefits. The decline in physical activity that occurs during adolescence may be occurring much earlier, in childhood, and no clear evidence that adolescent declines in physical activity were substantially greater than during childhood [9,10]. A comparative study of 32 countries in Europe and North America showed that most adolescents do not meet the physical activity recommendations [11].

The levels of physical activity vary between countries [11], and Lithuania has a high prevalence of physical inactivity in children and adolescents. Evidence from the 2001/2002 Health Behavior in School-aged Children (HBSC) survey found that 42.7% of Lithuanian adolescents reported being physically active [12]. The comparative study mentioned above found that, of the 32 countries, the most significant decrease in MVPA from 2002 to 2010 was in Lithuania [11]. A recent nationally representative study showed that 66.6% of Lithuanian adolescents meet the physical activity recommendations but that physical activity levels decrease with age [13]. Although a decline in physical activity with increasing age has been observed [13], a recent HBSC survey reported that even 11-year-old Lithuanian children are less physically active (14% and 20% of the girls and boys, respectively) compared with the leading countries [14].

When focusing on the physical activity of children and youth, it is important to consider physical activity not as a single construct, but as a composite of different components—as structured (e.g., participation in a sports team or club) and unstructured (e.g., play in free time). A relationship between participation in structured physical activity and positive development outcomes, such as better physical performance, academic achievement, and aspirations, has been established [15]. Negative relationships between structured physical activity and mental health problems, such as depression, anxiety, and aggressiveness, have also been reported [16]. Longitudinal studies have shown that greater participation in organized sport is associated with increased participation in free play [17] and increased the probability of being physically active in adulthood [18]. These findings emphasize the importance of measuring both the overall physical activity level and its various components.

One of the beneficial outcomes of regular physical activity is improved physical fitness. This is important because of the global downward temporal trend in countries around the world [19] and in Lithuania in particular [20,21,22]. Given the decreasing participation in physical activity during adolescence, recent studies indicate the importance of physical fitness monitoring [23]. Physical fitness is associated with both mental and physical health [24,25] and is an important population health measure [26]. Despite the importance of monitoring physical fitness, many countries do not have surveillance systems in place [27,28]. Government-level commitment is necessary for developing strategic plans to increase the levels of physical activity and physical fitness.

To understand better the scientific evidence on physical activity of children and youth, it is important to provide regular, country-level, and comprehensive analysis of the scientific evidence. The Active Healthy Kids Global Alliance produced the first Global Matrix (*Global Matrix 1.0*) to grade physical activity using a comprehensive, harmonized “Report Card” framework in 15 countries in 2014 [21]. Two years later, the *Global Matrix 2.0* presented the results from 38 countries [28]. In 2018, the *Global Matrix 3.0* results, based on the involvement of 49 countries, were released. Lithuania participated in the *Global Matrix 3.0* and produced its first Report Card in 2018. The Lithuanian Report Card included all available evidence related to 10 indicators of the physical activity levels of children and youth.

The purpose of this article is to summarize the process and results of the first Lithuanian Report Card (2018 Lithuanian Report Card on Physical Activity for Children and Youth). The novelty of this article is that it: (a) presents the first Lithuanian Report Card on the current state of the nation regarding physical activity levels of children and youth, (b) helps to identify gaps in current knowledge (research), (c) provides scientifically based evidence and recommendations to help researchers and stakeholders better promote physical activity for children and youth, and (d) provides comparisons with the other 48 countries that participated in the *Global Matrix 3.0*.

## 2. Materials and Methods

The development of the Lithuanian Report Card was initiated and coordinated by the Department of Health, Physical and Social Education, Lithuanian Sports University, in cooperation with the following partners: Vilnius University; Department of Physical Education and Sports under the Government of the Republic of Lithuania; National Olympic Committee of Lithuania; Ministry of Health of the Republic of Lithuania; and Ministry of Education and Science of the Republic of Lithuania. In addition, an international expert mentor (Dr. Jaak Jürimäe from University of Tartu, Estonia), and leaders of the Active Healthy Kids Global Alliance were involved in guiding the development of the Lithuanian Report Card. The development of the Lithuanian Report Card adhered to the harmonized process explained in detail elsewhere [27].

The Lithuanian 2018 Report Card included the 10 indicators endorsed by the *Global Matrix 3.0* for school-aged children and youth aged 5–17 years. These indicators represent individual behavioral indicators (Overall Physical Activity, Organized Sport and Physical Activity Participation, Active Play, Active Transportation, and Sedentary Behaviors); sources and settings of influence indicators (Family and Peers, School, Community and Environment, and Government); and a health-related characteristic (Physical Fitness) [27].

The grades for each indicator are based on the percentage of children and youth meeting a defined benchmark using standardized definitions [27]. The grades were compiled according to the percentage of young people who met certain criteria. For example, the indicator Overall Physical Activity was graded according to the percentage of youth who performed ≥60 min of daily MVPA. A grade of A indicated that >80% of children and adolescents met this criterion. The other grades according to the percentage who met the criteria were as follows: B = well over half of children and young people (61–80%); C = about half of children and young people (41%–60%); D = some children and young people, but fewer than half (21–40%); F = very few children and young people (0–20%); and INC (incomplete) = no available data, the data were not considered to be truly reflective of the indicator, or a consensus on how to operationalize the indicator could not be reached. In addition, the percentages of children meeting the specific criteria were subclassified within each grade by assigning a plus or a minus (e.g., C+ reflected 54–59%, C 47–53%, and C− 40–46%).

The data used to inform the grading of each of the indicators among individual behavior indicators, personal characteristics, and settings and sources of influence are presented below and are briefly summarized in Table 1. The data used in the analysis were no older than 5 years (from 2013 to 2018).

### 2.1. Individual Behavior Indicators

The grade for Overall Physical Activity was based mainly on the three nationally representative studies [14,29,30], one research report conducted by the Hygiene Institute [31], and one survey representing the western part of the country [32]. The grade describes the percentage of children and youth who meet the physical activity recommendation of 60 min of MVPA per day on average. The grade for Organized Sport and Physical Activity Participation was derived mainly using data obtained from some of the same sources as used for Overall Physical Activity [30,32] plus one additional nationally representative survey [33]. This grade was assigned using data about children and youth participation in sport clubs or teams.

The grade for Active Transportation was based on several nationally representative surveys [30,33,34,35] and describes the percentage of children and youth who use active transportation to/from school. The grade for Sedentary Behaviors was based on three nationally representative samples [14,33,36] and two smaller-scale studies reporting data for the central and western regions of the country [32,37]. This grade describes the percentage of Lithuanian children and youth who meet the guidelines for recreational screen-based activities of ≤2 h per day [38].

### 2.2. Personal Characteristics

Physical Fitness was the only personal characteristic-related indicator. The results from a nationally representative study of 11–18-year-old schoolchildren were used to establish the Physical Fitness grade [22]. The generalized fitness indicator was developed by summarizing the results for the average percentile achieved in cardiorespiratory fitness testing of children and adolescents (20 m shuttle run, expressed as min or stages); lower-body muscular power (standing long jump, cm); upper-body muscular endurance (bent-arm hang, s); lower body muscular endurance (sit-ups, n/30s); and flexibility (sit-and-reach, cm). These results were compared with the European normative values for physical fitness in children and adolescents aged 9–17 years [23]. Details of the fitness testing protocols are available elsewhere [39].

### 2.3. Setting and Sources of Influence

The grade for Family and Peers was based on a number of small cross-sectional studies [40,41,42,43] and a nationally representative study [44]. These studies included family members and friends who encourage and support children’s and adolescents’ physical activity.

The grade for School was based on the Education Management Information System [45], a document from the Basic and Secondary Education Curriculum for Academic Years of 2017–2018 and 2018–2019 [46], and a parent survey [40]. The grade for Community and Environment was based primarily on nationally representative research, including municipality administrations [47], public health bureaus [48], and several surveys of children of different ages and their parents or guardians [29,30,35,49]. 

The grade for Government was based on several major policies and documents: Lithuanian Health Strategy 2014–2025 [50], National Sport Development Strategy 2011–2020 [51], National Public Health Care Program 2016–2023 [52], National Network of Health Promoting Schools [53], and the Olympic project for children and youth “Olympic Generation” [54]. The first three relate more to healthy lifestyle and physical activity funding and infrastructure, and the fourth added information about good practice for increasing physical activity. Data from the National Progress Strategy “Lithuania 2030” [55], Law on Physical Education and Sport of the Republic of Lithuania [56], Physical Education and Sports Support Fund [57], and General Programs for Primary and Lower Secondary Education [58] were also included.

### 2.4. Data Analysis

For each indicator, if data were available, the disparities (e.g., across age, gender, disability, ethnicity, socioeconomic status, region) and temporal trends were considered. In addition, the quality of evidence, sample size, and representativeness were discussed and, where possible, the most recent and larger studies were used throughout the grading process.

During the meetings held in February–April 2018, the initial grades were developed and adjusted to final grades during discussions, and consensus was reached on all grade assignments.

Comparisons were also made between Lithuania’s Report Card grades and those from other countries participating in the *Global Matrix 3.0*. In addition, we compared Lithuanian’s grade averages with those of very high human development index (HDI) countries and with those of nearby countries for some indicators.

## 3. Results

The 2018 Lithuanian Report Card is the first comprehensive assessment of physical activity behaviors, settings, and sources of influence for Lithuanian children and youth. It provides a synthesis of the best available evidence related to the physical activity of children and youth according to the harmonized report card methodology [27]. The grades for most indicators were from C− to C+. Family and Peers received a grade of D, and an INC grade was assigned for Active Play because of insufficient data. The grades for each indicator are shown in Table 2.

## 4. Discussion

### 4.1. Overall Physical Activity Levels

The grade for Overall Physical Activity for Lithuanian children and adolescents was C−. Based on self-reports, the amount of MVPA ranges from 1 to 2 h per day (33%) to >2 h per day (60.5%) in primary school children [30,32]. In comparison, among adolescents, fewer than 30% of boys and 20% of girls (age 11, 13, and 15 years) perform 60 min of MVPA daily [14]. Another study has also reported that 50% of youth from different municipalities of Lithuania meet the guidelines on at least 4 days a week [29]. The worst case was reported by the Hygiene Institute [31], which found that fewer than 10% of Lithuanian adolescent boys and girls exercise daily for ≥60 min. Although the reported levels of overall physical activity of primary schoolchildren may seem desirable, this indicator is rated as “less than satisfactory” because of the low adherence to the Global Recommendations on Physical Activity for Health in Youth [8].

As this is the first Lithuanian Report Card, we cannot compare these findings with those in previous reports. However, the *Global Matrix 2.0* study of 38 countries (including 24 countries with a very high HDI) in 2016 showed an average grade of D for Overall Physical Activity [28]. The *Global Matrix 3.0* comparative study showed, that among 30 very high HDI countries, the grade ranged from F to A− [59]. In addition to Lithuania, only two other countries (England and Hong Kong) received a grade of C−. Neighboring countries (Estonia and Poland) received a lower grade of D− for this indicator; that is, only 20–26% of children and youth meet the requirements for daily physical activity [27].

### 4.2. Organized Sport Participation

Organized sports have greater health benefits than non-organized physical activity because of the higher intensity compared with non-organized physical activity [60]. A grade of C was assigned for Organized Sport Participation. According to two national surveys on families with young children aged 6–9 years, 50–70% of primary school-aged children participate in sports or dancing clubs twice per week [30,33]. Another study revealed that 8–11-year-old children from the west of Lithuania spend on average 30 min/day on activities in sports or dance clubs, but only 11.3% of the respondents indicated that they participate in organized sports. The sports organized as extracurricular activities at secondary schools are chosen by 23.4% of schoolchildren [32]. The Lithuanian results are similar to the average of all countries who participated in *Global Matrix 3.0* (i.e., grade of C) [27], although the situation was slightly better (C+) among 30 very high HDI countries [59].

### 4.3. Active Play

Active Play involves symbolic activity or games with or without clearly defined rules. The benchmark for this indicator pertains to the percentage of children and youth who engage in unstructured/unorganized active play at any intensity for >2 h/day [27]. To provide data to assign a grade for Active Play, we identified three studies [33,34,36]. Unfortunately, these studies focused only on 6–9-year-old children playing outside for 2 h/day. Given the lack of data covering other age groups, this indicator was assigned an INC grade. We note that among all countries in the *Global Matrix 3.0*, more than half (29 of 49 countries) also graded active play as INC, and the average grade was D+ for those countries that did assign a grade [27]. There is a similar lack of studies about Active Play among the very high HDI countries: INC was assigned in 20 of 30 HDI countries [59].

### 4.4. Active Transportation

Active Transportation includes walking, cycling, skating, skateboarding, and any incidental activity associated with the use of public transport. A grade of C− was given based on studies showing that 45% of 7–8-year-old children use active transportation to get to school and 57.9% use active transportation to return from school [30,34]. Another study reported that 39.2% of 6–9-year-old children use inactive transport going to and coming from school [33]. Fewer youth and adolescents (aged 15–24 years) use active transport; for example, only 12% of them engage in activities such as cycling from one point to another on a regular basis [35]. Active transportation is closely associated with the use of public transport. For example, in rural areas in Lithuania, schoolchildren are often driven to school by school buses if their trip to school is >3 km. The percentage of children driven to school in the past 5 years has remained stable [61]. Active transportation has many health benefits [62]. The low use of walking or bicycling could be related to lack of sidewalks, crosswalks, and bikeways; lack of connectivity of pedestrian or bike infrastructure; and actual or perceived dangers of walking and cycling [63]. We note that the development level of a country is not related to the likelihood of children walking or cycling to school [59]. For example, eight of the 30 very high HDI countries were graded below C− and 11 of all countries in *Global Matrix 3.0* were graded below C− for Active Transportation [27].

### 4.5. Sedentary Behaviors

To grade sedentary behaviors, data on the time children spent watching TV/films, playing computer/video games, surfing the internet, and doing homework per day, both on weekdays and weekend days, were analyzed. A grade of C− was given for sedentary behaviors because 6–9-year-old children accumulate 2.6 h/day of screen time activities on average [36]. Similarly, 8–11-year-old boys and girls spend 2.2 h/day on screen-based activities in their spare time [32]. Furthermore, 74% of elementary schoolchildren spend >2 h/day of their free time using computers for playing games (other than homework) or watching TV at home or somewhere else [33]. More than 50% of those aged 11, 13, and 15 years report spending ≥2 h/day watching TV, and 40–51% report using a computer to play games in their spare time [14]. A study conducted in the central region of Lithuania indicated that more adolescents (aged 10–13 years) than younger children (aged 7–9 years) tend to spend time watching TV (16.2% vs. 10.8%, respectively) or use their computers other than for homework tasks (17.3% vs. 2.6%, respectively) for ≥3 h/day [37]. All 38 countries in the *Global Matrix 2.0* had an average grade of D in 2016 [28]. A more recent comparative study showed that among 30 very high HDI countries, the highest grade was B+, and the lowest was F [59]. Lithuania was among eight countries with a grade of C–. Among all 49 countries in the *Global Matrix 3.0*, 11 had a grade of C− [27].

### 4.6. Physical Fitness

Physical fitness plays a key role in a child’s healthy growth and development [64]. A grade of C+ was assigned based on nationally representative research conducted in 2012, which included 5099 11–18-year-old schoolchildren in 10 Lithuanian regions [22]. The EUROFIT test battery was applied to measure components of physical fitness [39]. The results showed an unsatisfactory level of physical fitness compared with published European normative values [23]. The percentiles achieved by Lithuanian children and youths compared with the European normative values for specific measures of physical fitness were as follows for boys and girls, respectively: aerobic capacity measured as cardiorespiratory fitness measured on the 20 m shuttle run test, 28.6 and 38.6 percentiles; explosive leg strength measured on the standing long jump, 65.7 and 62.9 percentiles; upper-body muscular endurance measured on the bent-arm hang test, 67.1 and 72.9 percentiles; abdominal muscle strength, measured as the number of sit-ups, 72.9 and 84.3 percentiles; and flexibility measured on the sit-and-reach test, 42.9 and 41.4 percentiles.

Physical fitness was lower in Lithuanian children and adolescents compared with European values and with the results for Lithuanian schoolchildren in the previous decades as measured in 2002 and 1992 [20,22,65]. There has been a substantial decline in flexibility, explosive leg strength, upper-body muscular endurance, and particularly aerobic capacity (cardiorespiratory fitness); for the latter measure, the number of completed stages decreased by nearly 50% in the past two decades [22]. By contrast, during the same time, there were improvements in abdominal muscle strength (sit-ups) in girls, agility (10 × 5 m shuttle run test) in boys, and balance (flamingo balance test, measured as the total number of falls in 60 s) in both boys and girls [22]. In comparison, for the other 48 countries in the *Global Matrix 3.0*, 27 lacked data (grade of INC), and only four of the other 22 countries had higher grades (A to B−) than Lithuania [27].

### 4.7. Family and Peers

Family and peers are among the most important sources of influence on participation in sports and physical activity. The grade for this indicator in Lithuania was D. There are differences between studies of the involvement of peers and family members (parents or guardians) in children’s and youth’s physical activity and their facilitation of physical activity and sport. More than half (54.8%) of Lithuanian adolescents report that they are often active with their peers [42]. However, only 36.6% of 15–17-year-old adolescents report that their parents encourage them to be involved in physical activity [42]. Only about 40% of primary schoolchildren’s parents (mostly mothers) are physically active enough [40,41]. Only 2.9% of primary schoolchildren’s parents exercise together, 5.7% take part in physically active leisure with their children on weekdays, and 14.3% and 33.8% of parents, respectively, on weekends [43]. Among adolescents, 17.6% indicate that at least one parent exercises regularly [44]. In the 2018 *Global Matrix 3.0* report, 17 countries had a higher grade than Lithuania [27], but 22 countries had a grade of INC. Among the 30 very high HDI countries, Lithuania was in 13th position with a grade of D [59].

### 4.8. School

The School indicator was graded C+. According to the Ministry of Education, Science and Sport, at least two physical education classes per week is a compulsory requirement for all schools of general education in Lithuania [46]. Additionally, 38% of Lithuanian schools belong to the National Network of Health Promoting Schools [40]. In lower (grades 5–10) and upper (grades 11–12) secondary schools, physical education classes are taught by physical education teachers, physical education in primary schools is taught by generalist schoolteachers [45]. All Lithuanian schools offer extracurricular physical activities. About half (51%) of schoolchildren’s parents agreed that sufficient numbers of extracurricular physical activities were provided in schools and that school gyms are available for exercising after school [40]. It is important to state that Lithuania is missing an official active school policy at the legislation level. Comparison of the Lithuania report card data with those from other 48 countries [27] showed that just four countries were assigned a grade of C+ and 26 countries were assigned a lower grade than Lithuania for the School indicator. Among the 30 very high HDI countries, Lithuania, with a C+ grade, was in 16–19th place [59].

### 4.9. Community and Environment

The grade for this indicator was based on policies related to physical activity at the local government level and the grade for this indicator was C grade. More than half (62%) of parents indicated that their neighborhood environment is safe [30]. Local sport clubs offer many opportunities for physical activity [49]. Most public health bureaus and municipality administrations indicated that they are implementing health-promoting activities or programs [47,48]. Although local administrators report implementing programs, only half of the parents agreed that the local authority or municipality was doing enough for its citizens in relation to physical activities [35], and they felt these authorities should take further appropriate action to increase in participation in physical activity [29]. The Lithuanian grade did not compare well to those of the other 48 country grades because 19 other countries achieved a higher grade [27].

### 4.10. Government

The benchmark for this indicator includes leadership, commitment, and allocation of resources to implement political strategies to promote physical activity. This indicator was graded C. This grade was based on several national documents that describe the country’s strategies for healthy lifestyles. Among them, in 2012 the Lithuanian government approved the National Progress Strategy Lithuania 2030, which focuses on health and healthy lifestyles but does not use the term “physical activity” [55]. However, steps have been taken as other important documents related to physical activity have been approved. In 2014, the Seimas of the Republic of Lithuania approved the Lithuanian Health Strategy 2014–2025 [50], which addresses the specific challenge of promoting physical activity. In 2011, the National Sport Development Strategy 2011–2020 [51] was approved by the Seimas of the Republic of Lithuania; one of the goals is to encourage public awareness that physical activity and sport have universal value and are prerequisites for a sustainable personality.

The National Public Health Care Programme 2016–2023 [52] was also approved with an emphasis to increase the physical activity of the population (especially in early childhood and pre-school institutions and schools), inform all people about the health benefits of physical activity, provide evidence-based knowledge and raise awareness of health-enhancing physical activity, encourage different groups within the population to choose appropriate physical activity, and reduce sedentary time. The grades ranged from F to A for the 49 countries participating in the *Global Matrix 3.0.* The average grade for Government was C, and eight countries received an INC grade [27].

In summary, the main Lithuanian policy documents do not prioritize health-promoting physical activity as a means of disease prevention and rehabilitation. Discussions about physical activity policy at the government level and its implementation are episodic, and guidelines for promoting physical activity in Lithuania are lacking.

### 4.11. Strengths and Limitations

Report Cards on Physical Activity of Children and Youth have been developed several times in several countries, though this is the first time this has been done in Lithuania. This first Report Card reveals the level of current knowledge about the physical activity levels of children and youth at the national level in Lithuania. Although this is the first attempt in Lithuania, the Report Card includes all 10 grades, and the assignment of grades was based on nationally representative data. It is particularly important that this study includes Physical Fitness and that this indicator assignment was based on nationally representative data because almost half of the other countries in the *Global Matrix 3.0* lacked the data needed to assign a grade for this indicator. However, some surveillance gaps remain for Lithuania to address; for example, the data on children’s active play are lacking. For some of the indicators, especially for Overall Physical Activity and Sedentary Behavior, grades were based on questionnaire survey studies and there were insufficient data from studies using objective measures (e.g., accelerometry). Most indicators were informed by cross-sectional studies. The grade for Community and Environment was based mostly on surveys of local municipality administrators and parents, and there is a lack of studies on children. Despite the aforementioned limitations, the 2018 Lithuanian Report Card provides the first comprehensive aggregation of the best available physical activity data, and identifies the main data gaps and problems particular to Lithuania.

### 4.12. Recommendations

The 2018 Lithuanian Report Card shows clear evidence on physical activity for children and youth, and highlights the challenges that need to be overcome. The following recommendations address physical inactivity in general with an emphasis on other behavioral and social influence indicators. Overall Physical Activity is rated as “less than satisfactory” because of the low adherence to global recommendations on physical activity for health in youth [8]. It is important to develop and implement a national-level physical activity strategy for schoolchildren, for example, by improving the physical education curriculum and teacher qualifications, integrating physical activity into the whole school day, making recess more active, and expanding outdoor physical education opportunities. Efforts to monitor the implementation of physical activity policy at schools and to explore the relationships with schoolchildren’s physical activity and other health indicators are also important. Strategies such as active breaks could be implemented, especially to target those who are most inactive.

Methods to address the social influences on the physical activity of children and youth include motivational social support for families at an organizational level (school and workplaces) and community level (community leaders). To improve physical fitness, a national fitness monitoring system could be established to facilitate data gathering and dissemination procedures to enable the results to be available to those directly responsible for children’s physical development and health. Timely steps to improve physical fitness could be taken. Data analysis based on fitness monitoring will contribute to the improvement in public health, reduction in health care cost, and public welfare in general. In practice, physical fitness monitoring in school-age children will be useful for developing ways to meet schoolchildren’s needs and to organize informal physical education more effectively, provide guidance to authorities about formulating policies to promote physical activity, conduct evidence-based interventions, apply effective health-enhancing educational programs, guide educators and parents, and track physical fitness changes with time. Finally, collaboration among researchers and policy makers is crucial for taking the necessary steps towards improving physical activity indicators among children and youth.

## 5. Conclusions

The first Lithuanian Report Card on Physical Activity of Children and Youth shows that Lithuanian children and youth have less than satisfactory levels of organized physical activity, active transportation to and from school, community and built environments, and government strategies and investments. The low levels of support from family and peers require more attention from health promoters. There is a gap in the evidence about active play that should be addressed by researchers and policy makers. Periodic replication of the Lithuania Report Card is encouraged because it provides a summary of recent scientific evidence that should encourage actions to increase the chance for improvement. It will be important to review the results of physical activity and related indicators in Lithuanian school-age children and to monitor these continuously at the national level. This will help contribute to the analysis of general health-related information in Lithuania and programs to predict health risks. The data collected should be compared with those from other countries to provide a broader picture of the situation throughout Lithuania.

## Figures and Tables

**Table 1:** The main data sources and relationship to indicators.

Data Source	Methods, Subjects’ Age, and Sample Size	Variables and Their Contribution to Physical Activity Indicators (1–10) *
WHO European Childhood Obesity Surveillance Initiative (COSI study)	National representative survey, Lithuanian data: parents of 1st graders (n = 3802) [30,34] National representative survey, Lithuanian data: parents of 6–9-year-olds (n = 4436) [33] National representative survey, Lithuanian data: 7-year-old, (n = 4955) [36]	- Time spent by children in physical activity of moderate-to-vigorous intensity (in hours/day) (1) - Participation in organized sports at least twice a week (2) - Parents indication about safety of their neighborhood and areas for play and exercise (9) - Adolescents transportation to/from school (4) - Participation in organized sports at least twice a week (2) - Screen time spending < 2 h per day (5) - Adolescents transportation to/from school (4) -Screen time (5)
Health Behavior in School-aged Children (HBSC) study	National representative survey: 11-, 13-, 15-year-olds, n = 5730 [14]	- Fulfilment of recommendation related to physical activity (60 min of MVPA/day, 7 days per week) (1) - Screen time spending < 2 h per day (5)
Health Behavior in School-aged Children (HBSC) adapted study	Survey in 6 municipalities of Lithuania: parents and guardians of 13–14=year-olds (n = 2962) [29]	Fulfilment of recommendation related to physical activity (60 min of MVPA/day, 7 days per week) (1) - Adults perceiving municipality doing a good job at promoting physical activity (9)
Report on lifestyle of school-aged children	National representative survey: 11-, 13-, 15-year-olds (n = 38,633) [31]	- Fulfilment of recommendation related to physical activity (60 min of MVPA/day, 7 days per week) (1)
Organizing leisure time in a family	Survey in Western Lithuania: 8–11-year-olds (n = 614) and their parents (n = 604) [32]	- Time spent by children in physical activity of moderate-to-vigorous intensity (in hours/day) (1) - Participation in exercise-based extracurricular activities (2) - Screen-based activities to be less than 2 h per day (5)
Physical fitness study	National representative physical fitness test, 11–18-year-olds (n = 5099) [22]	- All students undergo tests: endurance (20 m shuttle run) (min/stages), lower body muscular power (standing broad jump) (cm), upper body muscular endurance (bent arm hang) (s), lower body muscular endurance (sit-ups) (n/30s), flexibility (sit-and-reach) (cm).
Study on lifestyle among 7–17-year-old children in Lithuania	Survey in 40 schools of Kaunas region: 7–9-, 10–13-, and 14–17-year-olds (n = 3990) [37]	- Screen time (5)
Physical activity, physical capability, nutrition habits,	Local survey, 1st–4th graders (n = 135) [41]	- Parents being physically active (7)
Family and peers influence study	Local survey, 15–17-year-olds (n = 400) [42]	- Family members who are physically active with their kids (7) - Family members facilitate physical activity opportunities for their children (7) - Children and peers being physically active together (7)
Physical activity, socialization and physical education in Kaunas region and Greece	Comparative two country study, Lithuanian data, parents of 1st–4th graders (n = 159) [40]	- Parents being physically active (7) - Participation in after-school physical activity at school (8)
Health promotion activities implementation in local communities	National representative survey, Municipal Public health bureaus employee (n = 45) [48]	- Municipalities health promotion policies (9)
Parents’ attitude to physical activity study	Survey in Western Lithuania: parents of 9–10-year-olds (n = 349) [43]	- Family members who are physically active with their kids (7)
Lithuanian people physical activity study	National survey, 15–75-year-olds (n = 1519) [49]	- Adults perception of local infrastructure for physical activity (9)
Special Eurobarometer, Sport and Physical Activity	Survey among European Union countries, Lithuanian data, (n = 1023) [35]	- Adults perceiving their neighborhood as safe and evaluating local municipality efforts encouraging physical activity (9) - Youth and adolescents tend to use less active transport (4)
Practical application of physical activity promotion	National survey, municipality administrations (n = 32) and public health bureaus (n = 34), 2014 [47]	- Implementation of physical activity promotion intervention (9)
Ministry of Education, Science and Sport	Basic and Secondary Education Curriculum for Academic Years [46]	- Regulation for at least two physical education classes per week (8)
Education Management Information System	Requirement for physical education teachers [45]	- Physical education classes are taught by physical education teachers, physical education in primary schools are taught by primary school teachers (8)

* Indicators: (1) Overall Physical Activity, (2) Organized Sport Participation, (3) Active Play, (4) Active Transportation, (5) Sedentary Behaviours, (6) Physical Fitness, (7) Family and Peers, (8) School, (9) Community and Environment, (10) Government. MPVA—moderate-to-vigorous-intensity physical activity.

**Table 2:** Grades according to physical activity indicator in the 2018 Report Card on Physical Activity for Children and Youth.

Indicator	Lithuania Grades	Global Matrix 3.0 Average Grade for the 49 Countries	Global Matrix 3.0 Average Grade for very High HDI Countries
Overall Physical Activity Levels	C−	D	D-
Organized Sport Participation	C	C	C+
Active Play	INC	D+	D+
Active Transportation	C−	C	C−
Sedentary Behaviors	C−	D+	D+
Physical Fitness	C+	C−	C−
Family and Peers	D	D+	C−
School	C+	C	C+
Community and Environment	C	C	B−
Government	C	C	C+
